# The experience of interpreter access and language discordant clinical encounters in Australian health care: a mixed methods exploration

**DOI:** 10.1186/s12939-018-0865-2

**Published:** 2018-09-24

**Authors:** Jennifer White, Trish Plompen, Christian Osadnik, Leanne Tao, Emily Micallef, Terry Haines

**Affiliations:** 1 School Primary and Allied Health Care, Monash University, Melbuorne, VIC Australia; 2 Partnerships & Service Design, Monash Health, Melbuorne, VIC Australia; 3 Monash Lung and Sleep, Monash Health, Melbuorne, VIC Australia; 4 School of Primary and Allied Health Care, Monash University, Kingston Centre, 400 Warrigal Rd, Melbuorne, VIC 3192 Australia

**Keywords:** Inpatient medicine, Access to care, Language discordance, Qualitative

## Abstract

**Background:**

Current evidence highlights that language discordant clinical encounters seriously compromise patient quality of care and health outcomes. We aimed to characterise patterns of interpreter service use in medical inpatient wards use and explore clinician experience of language discordance.

**Methods:**

Participants included medical students, residents, attending physicians, nursing and allied health professionals working in General Internal Medicine wards across two tertiary referral hospitals servicing a large Australian health care area. This study involved a retrospective electronic medical record audit of interpreter use. Six focus groups were conducted with 32 participants. Data were analysed using an inductive thematic approach with constant comparison.

**Results:**

Allied health professionals were identified as the largest users of interpreter services, followed by medical doctors. Distinct themes emerged regarding clinician experiences of language discordant encounters including: (1) Negotiating care when unable to get an accurate assessment; (2) Over servicing to fill in the gaps; (3) Using family members instead of professional interpreters: a vexed solution; (4) Disparities in care provision; and (5) Communication drought: broken by a flood.

**Conclusions:**

Patients with low English proficiency are at risk of being less informed of care processes, and having a very large volume of information given in a shorter period of time when an interpreter is present. There is a need for systematic and transformative change that addresses utilisation of professional interpreters as well as embedded healthcare culture and practices leading to less interaction with patients with limited English proficiency and reliance on family members as informal interpreters.

## Background

Language discordance occurs when a patient, carer and/or health care professional lack proficiency in the same language. This is an issue for the national and international health care systems due to increasing cultural diversity in terms of country of birth, languages and religious affiliation [1, 2]. The most recent Australian census showed that nearly half (49%) of all Australians were either born overseas (first generation) or had at least one parent born overseas (second generation) [3]. Likewise, the most recent Canadian census showed that one fifth (21.9%) of the population were foreign born and in the United Kingdom, 1 in 7 (14%) were foreign born abroad and 1 in 11 (9%) had non-British nationality [4]. As a result many people may not be able to speak, read, write, or understand the English language at a level that permits them to interact with the health care system [5, 6]. Reducing language and cultural barriers in healthcare are significant factors in resolving health disparities [7, 8].

Evidence show that patients with low English proficiency (LEP) who experience language discordant clinical encounters have poorer quality of care and health outcomes [9, 10]. Documented experiences of patients with LEP experience include: reduced access to care, fewer physician visits and subsequent reduced comprehension of diagnoses and treatment; medication complications, diagnostic errors and discharge from hospital with poor understanding of discharge [9–12]. Growing evidence also highlights that patients with LEP express dissatisfaction with their health care, and are less likely to adhere to crucial follow-up care [13, 14]. Clinical uncertainty due to language discordant clinical encounters can lead to additional costs to the health care system such as increased diagnostic testing and increases in length of hospital stay [15, 16].

The Victorian Language Service Guidelines has been developed to provide guidelines that identify when language services should be offered to clients based on legislative requirements and best practice service delivery [17]. This is in line with evidence that the use of professional interpreters, including access to sign language interpreters, has been to shown to improve health care quality and satisfaction with care [18], however variability exists in access to professional interpreters even when services are available and accessible [19]. Within the context of this study, one Australian study found that patients with LEP had only a one in 100 chance of having a professional interpreter engaged when required in a primary care setting [20]. Instead health services frequently used inexperienced, family members, bilingual staff or non-medical staff to overcome language barriers and/or reduced access to professional interpreters is to use medically [21]. Use of non –accredited interpreters can compromise quality of care when people do not have sufficient skills to interpret health and medical terminology [21], symptoms and treatment plans sufficiently to allow participation in medical decision making [13, 22, 23]*. Furthermore, health care professionals and p*arents have been shown to rely on their children to provide interpretation in medical settings. Relying on children as interpreters may result in emotional distress due to exposed to sensitive, confidential and complex information, which can comprise their well-being [24]. Indeed previous research in the deaf community has highlighted a reliance on children to interpret for Deaf parents, in the absence of access to sign language interpreters, and the subsequent implications raised in this community might pertain to the wider population with language discordance [25, 26].

In Australia, general medical physicians provide specialist inpatient services across a spectrum of health and illness not limited by the boundaries of medical subspecialties. However, there have been few Australian studies documenting clinician experiences of language discordance, and access to interpreters despite the health risk to many patients with LEP. This research aimed to answer the following questions: (1) When, why and by whom are interpreter services being requested and used within general medicine wards, and (2) what are clinician experience of language discordance, access to and use of interpreters when treating patients admitted to medical inpatient units.

## Methods

### Setting

Monash Health is the largest public health service in Melbourne, Australia responsible for servicing a region that is growing in population at the fastest rate in the region. Likewise, Victoria is also known for its diverse multicultural background, with healthcare system data demonstrating access by patients from more than 180 countries, speaking over 100 languages [27].

### Study design

This mixed methods study explored patterns of interpreter service use, and clinician experiences of language discordance, access to and use of interpreters when treating patients admitted to medical inpatient units. Firstly, a retrospective electronic medical record audit was conducted. Secondly focus groups were held with Monash Health clinicians with current and/or previous experience of work within the General Medicine program at the same hospitals.

### Participants and recruitment

#### Quantitative

A retrospective electronic medical record audit was conducted on 100 patients who accessed the interpreter/language service at either Monash Medical Centre (MMC) or Dandenong Hospital (DH) during the 2014–2015 financial year. Those admitted under the care of an acute medical unit were eligible for inclusion in the analysis. Cases were identified via a user-specific database that maintains records of all encounters provided by the Monash Health interpreter service. Only data relating to the first (index) hospitalisation per patient during the audit period were eligible for inclusion (i.e. no repeat admissions per patient). Multiple instances of service use within the index admission was eligible for analysis, if applicable.

#### Qualitative

Recruitment was open to Monash Health clinicians with current and/or previous experience of work within the General Medicine program at MMC and DH. Medical, nursing and allied health clinicians were invited to participate by response to an e-mail from the research team, based within Monash Health (via Program Directors). Detailed information was given about the study, and time and location of each focus group in order to access as many interested clinicians as possible. In some cases, Department Heads nominated staff depending on staff availability on the day. All participants provided written informed consent.

### Data collection

#### Quantitative

All data were extracted directly from hospital electronic medical records by one assessor using a standardised template. Random checks of accuracy were performed by a second independent assessor to verify data. Data were extracted regarding the timing of documented non-English speaking background status, timing and frequency of family-assisted interpretation, reasons for interpreter use and the healthcare professions that accessed the interpreting service.

#### Qualitative

Six focus groups (each composed of 5–8 participants) were conducted. Three were conducted at both MMC and DH (1 included medical officer participants; and 2 included nursing and allied health participants at varying times in order to promote inclusion of staff working part-time and differing shifts). A moderator and experienced qualitative researcher (JW) conducted the focus group and one facilitator (TP) took detailed notes that informed continued data analysis. The moderator was not associated with the implementation of the interpreter service; this assisted to reduce bias and facilitated open discussion that allowed participants to express their opinions. A schedule of questions (see Table 1) guided discussion about clinicians’ experience of language discordance.

**Table 1:** Focus Group Interview Guide

Question	
Can you start by introducing yourself and giving a brief overview of your current position and how long you have worked in General Medicine?	
What is your professional experiences of working with non-English speaking patients? What is your professional experiences of using an interpreter or note?	
What is your experience of booking interpreters at Monash Health?	
When do you decided to use a professional interpreters? What are the triggers/key times where you would try to book an interpreter?	
Do you find there is a difference between using a professional interpreter versus using family/friends or other staff as ad hoc interpreters	
Are then any processes that could be changed on the ward to facilities use of an interpreter	
Do you think it is important for a patient to have access to an interpreter?	
How are a patient’s interpreter requirements communicated in your area of work?	

### Data analysis

#### Quantitative

Characteristics related to interpreter service use included data regarding the number of interpreter encounters, timing of interpreter use (early / middle / late during the admission), use of family members for translation (where documented), health professions that accessed the service, mode of interpretation (in-person vs phone) encounters. These data were inspected for normality then evaluated via summary statistics appropriate to normal or non-normal distribution and represented visually via pie chart or column graph.

#### Qualitative

Focus group data were audio-recorded with participant consent and transcribed verbatim, with identifying data removed. The inductive analysis process included the three types of constant comparative method [28]. This involved: (i) identifying units of meaning using a process of reading the transcripts line-by-line; (ii) grouping units into categories whereby each category was labelled to assist with retrieval between the data; and (iii) examining relationships between codes in the context of the research question in order to form themes. To ensure accuracy, consensus coding was undertaken by two researchers (JW and TP). Any differences in researcher perspective were resolved by negotiation and, if necessary regrouped and recoded until consensus were reached. Lincoln and Guba’s [29] work guided the analysis process to ensure rigor, through the strategies of credibility, transferability, reliability and conformability.

## Results

### Quantitative

**Fig. 1:**
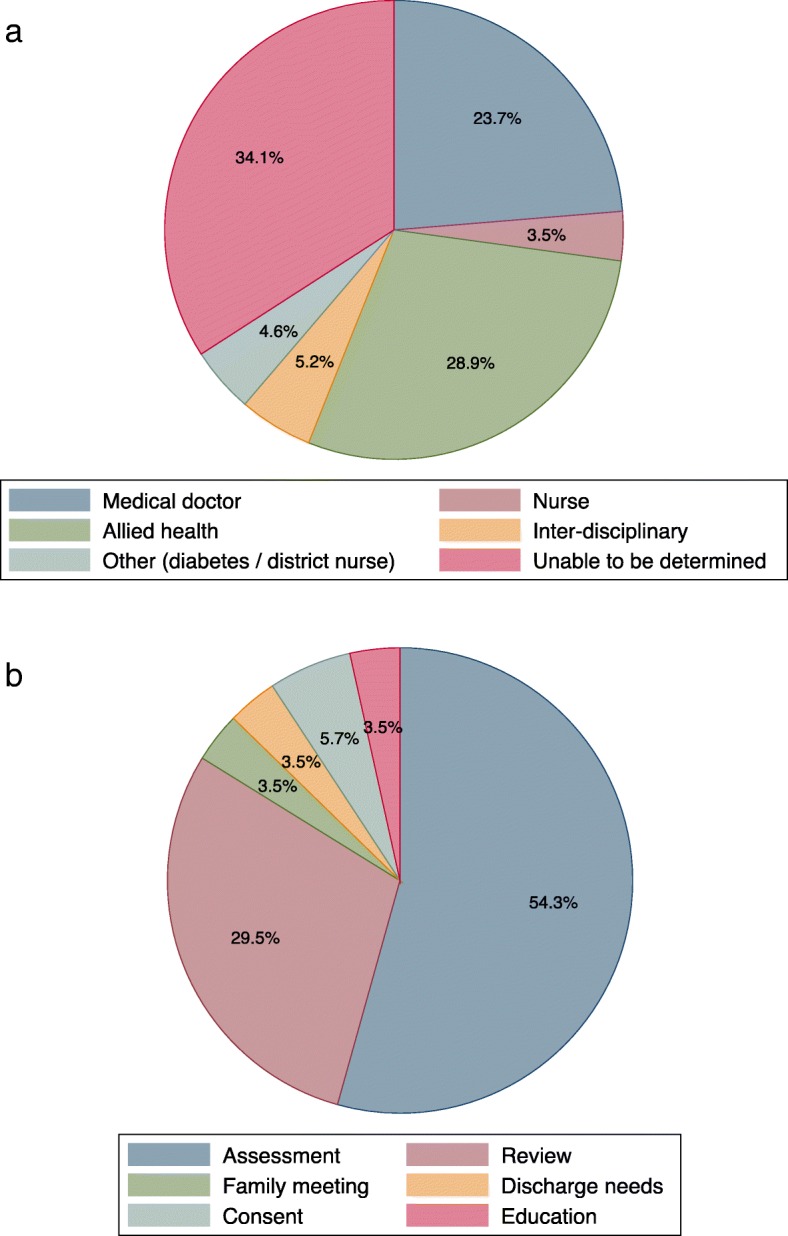
**a**. Users of interpreter service, by profession. **b**. Reasons for interpreter access, by purpose

One hundred patients (mean age 80.3 (10.2) years, 54 (54%) male) representing 211 interpreter episodes were identified via the interpreter database during the reference period. Use of interpreters was generally low on a per-patient basis (median 1, range 1–12) and allied health professionals were identified as the largest user of the interpreter service, followed by medical doctors (Fig. 1a). The most common reason for booking interpreters was for patient assessments and patient reviews (Fig. 1b).

**Fig. 2:**
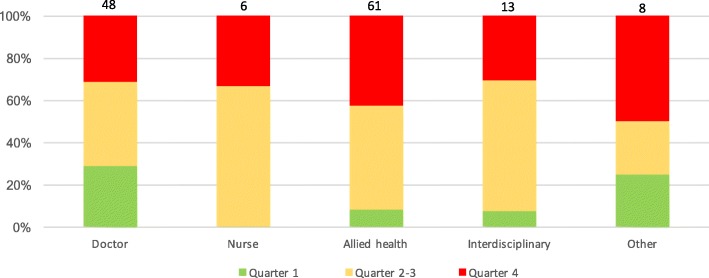
Distribution of interpreter encounter timing (by length of stay quartiles), according to healthcare professional categories. Green shading denotes encounters that occurred during the first quartile of the admission; yellow denotes encounters during the middle two quartiles of the admission; red denotes encounters that occurred during the last quartile of the admission

The timing of health professional use of interpreter services is summarised in Fig. 2. Most of the encounters occurred early within the hospital admission. A majority of these early encounters and were initiated by medical doctors, typically for initial patient assessments in the emergency department.

Use of family members as interpreters occurred in at least 49% of included patients during their inpatient stay (median encounters 0, range 0–13).

### Qualitative

Participant demographics are shown in Table 2.

**Table 2:** Focus group participant demographics

Participant Characteristics *N* = 32	No.
Age	
21–25	1
26–30	12
31–35	7
36–40	2
41–45	4
46–50	1
51–55	1
56–60	2
60+	1
Unknown	1
Educational Level	
Bachelor Degree	22
Certificate/Diploma	4
Post Graduate	4
Doctoral Degree	2
Years in practice	
1–4	4
5–9	13
10–14	8
15–19	3
> 20	3
Unknown	1
Discipline	
Allied Health	15
Medical	8
Nursing	7
Support Services	2

Five distinct themes emerged regarding clinician experiences of language discordant encounter. These included: (1) Negotiating care when unable to get an accurate assessment; (2) Over servicing to fill in the gaps; (3) Using family members instead of professional interpreters: a vexed solution; (4) Disparities in care provision; and (5) Communication drought: broken by a flood.

1. *Negotiating care when unable to get an accurate assessment*

Doctors reported being unable to get an accurate or complete medical history of patients with LEP admitted to General Medicine due to language barriers. Doctors identified barriers to assessment where there had been no previous interpreter involvement or handover had been insufficient, resulting in the need to repeat an initial assessment.

“Not everything has been documented or handed over – so you take the same history again. So it is often a double up, whereas a lot of other specialities surgical, medical, have just one team that you see from start to finish.” (DH, medical FG)

When taking a medical history, doctors described their approach as ‘taking the path of least resistance’, especially when faced with the extra time ascribed to treating patients with LEP in the context of a large workload. In the first instance doctors reported they commenced taking a medical history and/or treating acute symptoms without an interpreter or talking to the patient.

“So often these patients are having these blood tests done or having tests done or having antibiotics and they have no idea why.” (MMC, medical FG)

Doctors spent extra time performing alternate activities such as: checking admission summaries and/or previous medical reports and General Practitioner (family physician) letters to verify information, and inferring information from tests and other empirical data.

“When the patient is not able understand us and we cannot understand the patient … often we are doing a lot of empirical therapy based on external things to their history.” (MMC, medical FG).

Alternatively doctors indicated they tried to communicate with patients using non-verbal cues (e.g. gestures, facial expressions), or family members to translate. This meant that decisions were made in the absence of being able discuss and clarify symptoms with the patient. All doctors reported they were “Used to dealing with not knowing the full story and knowing that we don’t know the full story” (DH, medical FG) resulting in diagnoses being made and treatment plans instigated without an accurate medical scenario.

2. *Over servicing to fill in the gaps*

In the absence of a complete medical history and immediate access to a professional interpreter to assist communication with patients, doctors reportedly ordered more diagnostic tests to assist obtain a clear medical history. Delays in interpreter access were reportedly greatest when booking an interpreter late in the day or when requiring an interpreter for a less common language, that need to be out-sourced. In some cases it took several days to outsource a rare language.

“We default to just ordering more tests rather than waiting to get an interpreter.” (DH, medical FG)

Doctors readily justified over investigating as being a more efficient means of obtaining an accurate medical history in contrast to taking extra time to organise an interpreter and talk to the patient.

“I think there is still some clinician’s perception that, for their work efficiency, it’s easier to fill in a form and request a test than it is to organise an interpreter.” (DH, medical FG)

“If you don’t have the ability to communicate with the patient, it’s really hard to even start and we end up over-investigating… and start acting on the results rather than on the patient’s symptoms.” (DH, medical FG)

When probed further, doctors readily acknowledged that while they were comfortable working with limited information, there was also potential for error.

“So, it’s more about being comfortable with that [acting without the full picture] and …the feelings of risk.” (DH, medical FG)

On further reflection, doctors expressed that such scenarios ideally required a decision support system.

“I just think we really don’t have any structure, so that at least we know when we are within the bounds of safety and when we are actually nearing the boundaries - and we definitely have to be more conscious about when we have to call for help.” (DH, medical FG)

3. *Using family members instead of professional interpreters: a vexed solution*

Doctors complained that the electronic system in place to request interpreter services at their hospital was not user friendly, being ‘clunky’ and a disincentive to requesting an interpreter. All clinicians also stated that the use of an interpreter added significant time to their consultation, adding to this disincentive. To speed processes clinicians indicated that they frequently relied on family members to translate for patients with LEP.

“We rely on family quite a bit ….obviously they are very useful for us because we can get quick information conveyed to the patient and also back from the patient. Not that we wouldn’t use [professional] interpreters but I guess it [using the family] makes it a bit more efficient.” (MMC, medical FG)

The use of family as interpreters was readily perceived to introduce bias and clinicians recognised that family members may interfere by interpreting incorrectly, provide incomplete information or withhold information.

“We use it [family members] with the knowledge that we do not know what is being said, so we do not actually know whether the questions we are asking are being relayed or if it is the family member’s interpretation of the information or the question.” (MMC, medical FG)

Likewise all clinicians indicated that it could not be assumed that family members were proficient in English despite asking them to interpret or partake in decision-making. There was also scope for misunderstanding when families appeared to understand through agreeable gesturing such as nodding and smiling. However, all clinicians found it more convenient to use family members as they were more readily accessible and, unlike professional interpreters, were able to provide additional contextual information.

“They know their mum and dad better than us, so cognitively, they can quickly tell us is the patient is normally liked this at home or do they seem more confused.” (DH, medical FG)

All clinicians reported that there were an increasing number of clinicians who spoke more than one language to varying levels of fluency. As a result, collaboration with bilingual colleagues was frequently sort, perceived as an effective and convenient communication strategy.

“That is the easiest thing you face with a non-English-speaking patient, just look around your team and see if someone speaks the language. Sweet! [Great to hear] You’re up!” (DH, medical FG)

However bilingual staff reported mixed feelings towards adopting an interpreter role as they felt they were not skilled enough to interpret complex medical terminology.

“I would prefer an interpreter there, even if I [as a doctor] speak the language.” (MMC, medical FG)

4. *Disparities in care provision*

Clinician reports suggested that patients with LEP received less than optimal care as compared to English speaking patients and this was a readily accepted norm in daily practice. Likewise, there was consensus that the need for an interpreter was determined by staff perceptions of needs rather than patient expressed needs.

In many instances patients were transferred to the ward outside of business hours and admitting clinicians undertook admission and orientation of the patient to the ward without use of a common language or interpreter. Instead accompanying family members were often relied upon to complete admission information.

“To be honest the first point of call [during after hours admission] is the family” (DH, medical FG)

Ward rounds were perceived as central to care planning being a critical time to monitor patients and act quickly towards ordering necessary investigations in a timely fashion, “Because everything [diagnostic departments] seems to shut down after 2 o’clock in the afternoon;” (MMC, medical FG) as well as not to be delayed in their outpatient clinics. As a result doctors reported they were reluctant to have early morning ward rounds prolonged by the inclusion of professional interpreters. Instead, the period of time after the ward round was stated to be a more appropriate time to clarify information by talking with patients and family members and access an interpreter. Arranging an interpreter was typically delegated to junior doctors (intern or registrar) which meant that patients with limited English proficiency seldom had the opportunity to talk directly to, or ask questions, of the doctor (consultants) with the most expertise, something that usually happened during ward rounds.

Lack of time and difficulty coordinating consultations with a interpreter or family member meant that key conversation towards diagnosis and treatment were lacking. As a result the patient and their family had potential to be less informed of care processes since interpreters were primarily booked “For something important like a family meeting, but not just for diagnosis.” (MMC, medical FG).

5. *Communication drought: broken by a flood*

Clinicians readily reported they spent less time with the patient with LEP in comparison to English speaking patients.

“Patients who speak English always get the constant communication such as, “okay your diagnosis is this, our expectation of you is this, the expectation of us is this, the results are going to be blah blah” when they are going to come out. But for people who do not speak English they don’t get that.” (MMC, allied health FG)

Day to day communication was difficult and all clinicians reported that they ‘saved’ their information for when an interpreter had been booked. The consequence of this was that patients received a lot of information in one interpreter session, maybe from one or more clinicians.

“We will wait to get the interpreter because we know we are going to get the CT or the MR or ultrasound or the lab results back so that we can then batch all of the results that we have got at one time and have one big conversation with the interpreter rather than using them more frequently for very short interactions.” (DH, medical FG)

All clinicians perceived chronic disease education to be an essential component of effective preparation for discharge in general medicine, and expressed concern regarding the capacity of patients to recall the large amounts of information provided quickly and in a short space of time. In contrast, English speaking patients were reported to receive the same information over several sessions and have their knowledge checked.

“For an English patient, we might tell them the same piece of information three days in a row because that is how long it takes for them to absorb. If you have only got an interpreter for 30 minutes and it took you two days to get the interpreter, you don’t have that luxury and so they are more likely to re-present because they don’t completely understand what is going on.” (DH, allied health FG)

Discharge planning was another scenario where communication gaps were common due to fewer clinician interactions. Many cultures valued caring for an elder at home and took a longer time to accept the need for rehabilitation or high level care.

“The family was like, how we are going to send our mum to a nursing home or somewhere else? So that actually took a very long time for them to decide.”

(DH, allied health FG)

As a result delays in early family involvement often meant discharge plans needed to be changed or re-negotiated. Subsequently, extra time was required to have additional conversations and rebooking family meetings and interpreter to implement an alternative discharge plan, such as arranging community support and providing specific training (e.g. transfer and activities of daily living training) for family members to take a patient home safely.

## Discussion

This study demonstrated that language discordant clinical encounters resulted in the delivery of care where clinicians accommodated for a deficient clinical history by: ordering large numbers of tests; engaging family members or bilingual staff as pseudo-interpreters; not communicating regularly or providing key messages directly to patients; and when interpreters were present, provided a large volume of information in a short space of time. These rresults provide valuable insight into how clinicians can provide a better clinical service to patients from LEP. Further, clinicians accepted that clinical care given to patients with LEP was different to care given to English speaking patients.

Our findings echo with previous studies highlighting that language barriers between providers and patients result in poor quality care [30, 31].As immigration continues to increase across Western Countries, there is a greater need to address the linguistic needs of immigrant population to facilitate engagement with the health system in order to optimise care [32]. As a result it is important to provide opportunities for LEP patients to access to providers who speak their language [33]. While the provision of an interpreter service improves care, concern steams from evidence suggesting that the quality of provider–patient communication remains suboptimal [34, 35]. Ideally will greater diversity the health care work force will becomes more racially/ethnically and linguistically diverse and be able to assist to provided effective communication. It is important that the health care system utilises, train and/or recruit bilingual staff to meet the health care needs of an increasingly diverse population [32].

Quantitative data in our study showed that doctors’ were the highest users of professional interpreters during early hospital admission. While doctors made their best efforts to obtain an accurate medical history, qualitative data reveals that language discordance led to ongoing difficulties towards gaining a comprehensive early, accurate or complete medical history. Specifically results showed that doctors were more likely to allocate time towards collecting information from alternative sources, including ordering more tests and making decisions without talking to the patient or family.

Allied health were the largest users of the interpreter service however reports identified key gaps in service delivery with allied health staff spending less time with patients with limited LEP in contrast to English speaking patients. An additional, concerning finding in this present study stemmed from clinician reports suggesting that patients with LEP were less informed of care processes, and that a very large volume of information was communicated to patients in a shorter period of time when an interpreter was present. This highlights a significant barrier that conflicts with evidence that patients report a desire for increased participation and information sharing [36]. As a result, higher use of interpreter service among allied health did not equate with patient centred and better care. In fact all clinicians reportedly relied on family members as a means to communicate information despite clinicians’ recognition that this was suboptimal and potentially risky for the patient and organisation. The findings of our study suggest that having language concordance between providers and patients is still the optimal situation.

Bilingual staff were frequently perceived as being an effective and convenient alternative communication strategy due to delays in accessing an interpreter for certain language, especially rare languages, and barriers to booking an interpreter due to technical difficulties. However bilingual staff reported mix feelings towards adopting an interpreter role. As a result it is critical that bilingual staff are language certified and receive training in interpretation if they are used when medical interpreters are not present or are unavailable for the medical encounter. In this study context, the Victorian government policy states that people who cannot speak English need to be able to access professional interpreters where significant life decisions are concerned and when essential information is being communicated to enable people to make informed decisions about their lives [37]. However, having access to a professional interpreter is only the first step in overcoming the language barrier. We advocate for improved processes to promote greater effectiveness of the interpreter service once in attendance are needed to mitigate the potentially overwhelming effect of having multiple health professionals deliver a range of health information to patients in the short space of time that a professional interpreter is present.

### Strengths and limitations

This study generates important in-depth insight into patterns of use of professional interpreters. The strength of this study lies in access to a large sample of clinicians in a health service caring for a large culturally and linguistically diverse population. A broad range of experiences were identified and we achieved thematic saturation. However we did not capture the patient or family perspective.

### Future research

Future research should explore mechanisms for overcoming some of the barriers that clearly exist in patients being able to access and then effectively communicate with clinicians through professional interpreter services. This might include developing means for early identification of interpreter needs, transferring the role for ordering professional interpreter services from health professional staff to administrative staff, and trying to promote access to interpreter services early on during an admission so that the subsequent over-servicing can be averted.

## Conclusion

Patients with LEP are at risk of receiving fewer clinical interactions, being less informed of care processes, and having a very large volume of information given in a shorter period of time when an interpreter is present. There is an attitude that families be used as interpreters in the first instance. Greater access to and use of professional interpreters provides the opportunity for communication, reassurance and earlier evaluation and treatment where necessary.

## Abbreviations

DH: Dandenong Hospital
FG: Focus Group
LEP: Low English proficiency
MMC: Monash Medical Centre
